# CLEARPOND: Cross-Linguistic Easy-Access Resource for Phonological and Orthographic Neighborhood Densities

**DOI:** 10.1371/journal.pone.0043230

**Published:** 2012-08-20

**Authors:** Viorica Marian, James Bartolotti, Sarah Chabal, Anthony Shook

**Affiliations:** Northwestern University, Evanston, Illinois, United States of America; UCLA, United States of America

## Abstract

Past research has demonstrated cross-linguistic, cross-modal, and task-dependent differences in neighborhood density effects, indicating a need to control for neighborhood variables when developing and interpreting research on language processing. The goals of the present paper are two-fold: (1) to introduce CLEARPOND (Cross-Linguistic Easy-Access Resource for Phonological and Orthographic Neighborhood Densities), a centralized database of phonological and orthographic neighborhood information, both within and between languages, for five commonly-studied languages: Dutch, English, French, German, and Spanish; and (2) to show how CLEARPOND can be used to compare general properties of phonological and orthographic neighborhoods across languages. CLEARPOND allows researchers to input a word or list of words and obtain phonological and orthographic neighbors, neighborhood densities, mean neighborhood frequencies, word lengths by number of phonemes and graphemes, and spoken-word frequencies. Neighbors can be defined by substitution, deletion, and/or addition, and the database can be queried separately along each metric or summed across all three. Neighborhood values can be obtained both within and across languages, and outputs can optionally be restricted to neighbors of higher frequency. To enable researchers to more quickly and easily develop stimuli, CLEARPOND can also be searched by features, generating lists of words that meet precise criteria, such as a specific range of neighborhood sizes, lexical frequencies, and/or word lengths. CLEARPOND is freely-available to researchers and the public as a searchable, online database and for download at http://clearpond.northwestern.edu.

## Introduction

### Phonological and Orthographic Neighborhood Densities

In research on language, neighborhoods are a conglomeration of words that are highly similar to one another along a critical characteristic. Most commonly, neighbors are defined on the basis of shared linguistic features such as orthography, phonology, or semantics. Because a word’s neighborhood size (i.e., the number of neighbors it has; also called neighborhood density) can have an impact on a variety of linguistic tasks and processes, it has become an important psycholinguistic metric. However, in spite of the focus on neighbors in psycholinguistic research, neighbors are inconsistently identified, particularly across languages. These inconsistencies, which often arise as a result of researchers employing different databases, make it difficult to compare the effects of neighborhood density across studies. The current paper has two goals: (1) to introduce a centralized database of neighborhood information for five commonly-studied languages – Dutch, English, French, German, and Spanish – and provide a single corpus through which neighborhoods can be indexed cross-linguistically; and (2) to compare general properties of neighborhoods across these five languages using this database in order to determine where and how languages differ in respect to their neighborhoods.

In the current paper, we examined two types of linguistic neighborhoods – orthographic and phonological. Orthographic neighborhoods are often defined according to Coltheart, Davelaar, Jonasson, and Besner’s [1] *N* metric, which refers to the number of words that can be constructed by substituting one letter of the target word. For example, the word *log* has *hog*, *lug*, and *lot* as orthographic neighbors. Phonological neighborhoods are calculated similarly, but instead of depending on grapheme substitution, phonological neighbors are constructed by substituting one phoneme of the target word [2]. *Fish* (/fι∫), for example, has *dish* (/dι∫/) and *fig* (/fιg/) as phonological neighbors. These “substitution neighbors” have historically been the focus of the literature and have dominated investigations of neighborhood size. However, research has also investigated the effects of addition (formed by the addition of a grapheme or phoneme, for example *and* has *hand* as an orthographic addition neighbor) and deletion (formed by the deletion of a grapheme or phoneme, for example *bend* has *end* as an orthographic deletion neighbor) neighbors [3].

The effects of phonological and orthographic neighborhood density on language processing have been well documented across a variety of tasks [4]–[11] and across multiple languages [12]–[15]. However, in spite of the prevalence of neighborhood effects, the nature of these effects is subject to debate. For example, neighborhood density may affect recognition and production processes differently [16], [17], and effects may vary depending on the language of presentation [13], [18], [19] (but see [14]). The ongoing debate surrounding neighborhood density effects, particularly across languages, underscores the need for resources that allow researchers to consistently identify orthographic and phonological neighbors across studies. For some languages, even the most basic descriptive data are not available, forcing researchers to continually recreate basic neighborhood and frequency statistics. Furthermore, even when descriptive statistics are available [13], [15], [20], [21], direct cross-linguistic comparisons are often not reported or possible.

While there have been some attempts to create consistent corpora from which neighborhood information can be derived, these corpora vary across languages. For example, N-Watch, a database of English neighborhood information [22], defines phonological neighbors according to the substitution of a single phoneme in any word position. BuscaPalabras, a database of Spanish neighborhood information [8], and E-Hitz, a database of Basque neighborhood information [23], define phonological neighbors according to those same rules, but also include words that differ by the addition or deletion of a phoneme from any word position.

**Figure 1:**
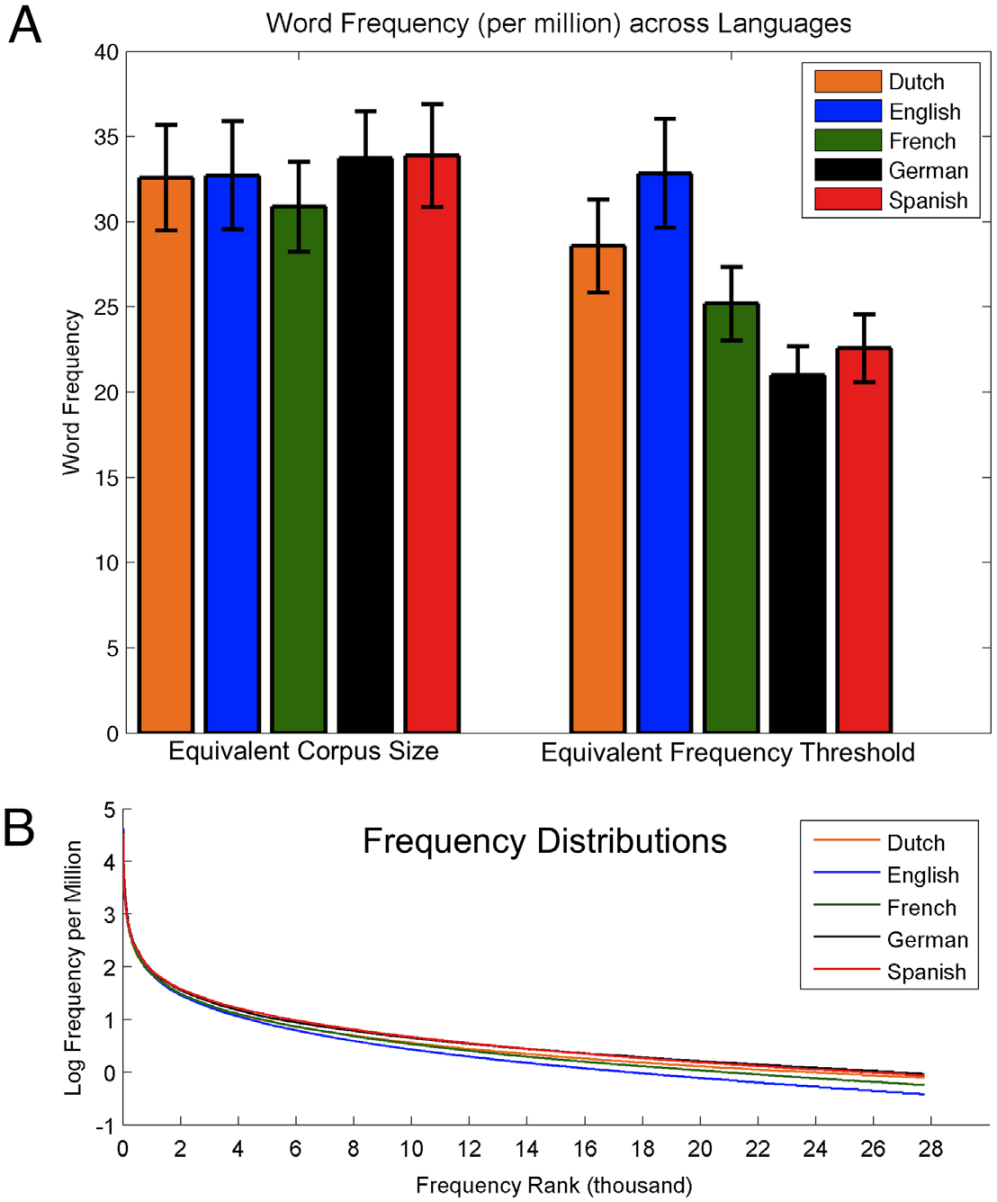
(a) Word frequency (per million) across Dutch, English, French, German, and Spanish. Equating corpus sizes (left) resulted in average word frequencies that were comparable across languages; size-equated corpora were thus used in all further analyses. If, instead, corpus size was defined only by a frequency threshold (right), differences in average word frequency emerged. (b) Word frequency distributions for each language, using equivalent corpus sizes.

The goal of this paper is therefore to introduce CLEARPOND, the Cross-Linguistic Easy-Access Resource for Phonological and Orthographic Neighborhood Densities, a catalog of neighborhood density across languages. Perhaps the most comprehensive psycholinguistic database to date is WordGen [21], which queries the CELEX and Lexique databases to provide searchable datasets for Dutch, English, German, and French. While WordGen controls for factors such as written word frequency, orthographic neighborhood size, bigram frequency, and word length, it is missing a number of relevant features including information on phonological neighbors, neighborhood frequency, and the ability to index neighbors across languages. The database that we present here has been controlled for word frequency to ensure that consistent and comparable tokens are sampled from each language, and provides data regarding word length, neighborhood density, and neighborhood frequency. We also provide measures of foreign neighborhoods (i.e., the number of Spanish neighbors of an English word, or English neighbors of a Spanish word, etc.) for use in bilingual comparisons. Neighborhoods are defined both orthographically and phonologically, with stimuli derived from film and television subtitle corpora that capture spoken word frequencies. Finally, we have defined neighborhoods by substitution, addition, and deletion. It is our intent that CLEARPOND will provide a standard from which neighborhood data can be easily extracted and that it will provide a comprehensive tool for psycholinguistic researchers.

**Figure 2:**
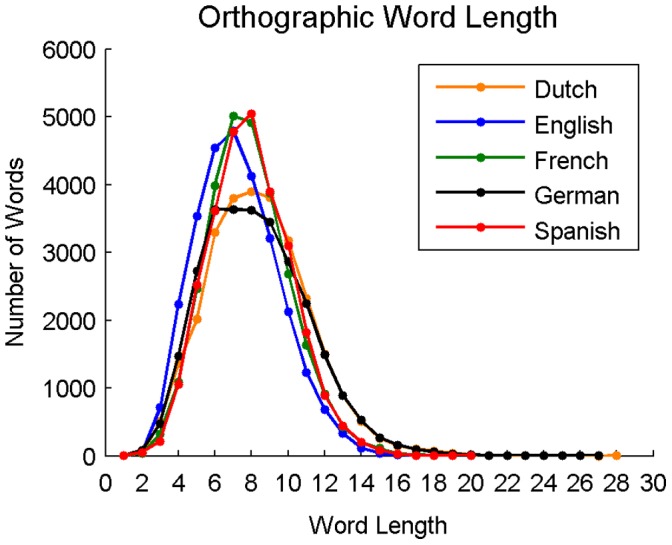
Distribution of orthographic word lengths for Dutch, English, French, German, and Spanish.

## Methods

### Selection of Corpora

To examine phonological and orthographic neighborhood densities across languages, we selected corpora for the following languages: Dutch (SUBTLEX-NL) [24], English (SUBTLEX-US) [25], French (Lexique) [26], German (SUBTLEX-DE) [27], and Spanish (SUBTLEX-ESP) [28]. Misspellings, including culturally-defined spellings (e.g., British “colour”), and foreign language intrusions (e.g., the English word “mind” in the Spanish corpus) were removed by cross-referencing each subtitle corpus with a dictionary in that language. Because all five corpora use the same source-material (i.e., film and television subtitles) to derive frequency data, they are highly comparable and well suited for cross-language comparisons. To increase similarity among the corpora, homographs were removed from the French corpus to match the parameters of the Dutch, English, German, and Spanish corpora (none of which distinguish between the different meanings of homographs). French homographs were reduced to a single entry, and the frequency per million of the collapsed entry was created by adding the frequency per million of each of the homographs. For example, the French word *est* is the third person singular form of the verb meaning “to be,” and has a frequency of 19,417 per million; *est* is also the French word for the cardinal direction East, which has a frequency of 81 per million. We collapsed these two entries into a single entry, *est*, that had a frequency of 19,498 per million.

**Figure 3:**
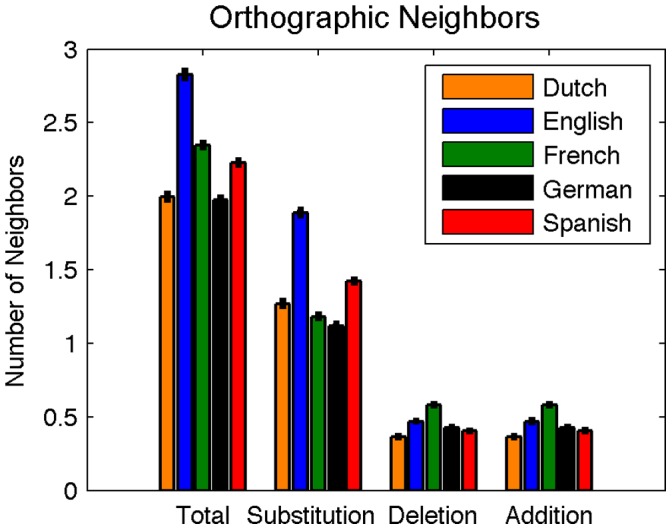
Mean orthographic neighborhood sizes for words in Dutch, English, French, German, and Spanish. Total mean neighborhood size (left group) includes single-letter substitutions (e.g., ‘log’ for ‘hog’), deletions (e.g., ‘end’ for ‘bend’) and additions (e.g., ‘hand’ for ‘and’).

**Figure 4:**
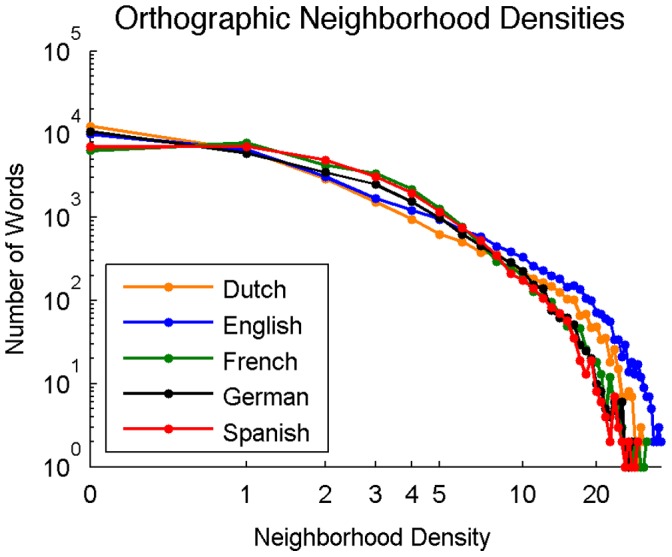
Distribution of orthographic neighborhood densities across Dutch, English, French, German, and Spanish (log-log scale).

Using large corpora (the subtitle lexicons range from 74,286 to 441,132 tokens) can lead to overestimations of neighborhood size compared to people’s actual working vocabularies. By only including words above a certain frequency threshold, the effect of very low frequency words (which are unlikely to be in people’s everyday, working vocabularies) on neighborhood calculations is reduced. In the present study, a frequency threshold of 0.34 per million was used, based on the standard used by Davis [22]. This frequency cutoff yielded a corpus size of 27,751 for English, which compares favorably to English vocabulary size estimates for educated adults (20,000 word families) [29]. However, the frequency cutoff yielded different corpus sizes across languages (Dutch: *N* = 31,691; English: *N* = 27,751; French: *N* = 34,113; German: *N* = 45,027; Spanish: *N* = 41,968), which would limit our ability to make cross-linguistic comparisons. Larger corpora are likely to inflate neighborhood size estimates, as a larger overall sample pool results in a larger pool of potential neighbor-candidates. To alleviate this concern, corpus size was equated across languages by including the 27,751 most frequent words in each language (based on the smallest corpus, English) in all further comparisons. Figure 1a (left) shows that when corpus size was equated, the languages had comparable average frequencies (Dutch: 32.58, *SEM* = 3.10; English: 32.72, *SEM* = 3.18; French: 30.87, *SEM* = 2.64; German: 33.74, *SEM* = 2.74; Spanish: 33.87, *SEM* = 3.02), while Figure 1a (right) indicates that the languages differed in average frequency when corpus size was instead defined by a frequency threshold. In addition, frequency distributions (Figure 1b) were comparable across languages when corpus size was equated. Together, these results provide support for the ability to make direct comparisons between the size-equated corpora.

**Figure 5:**
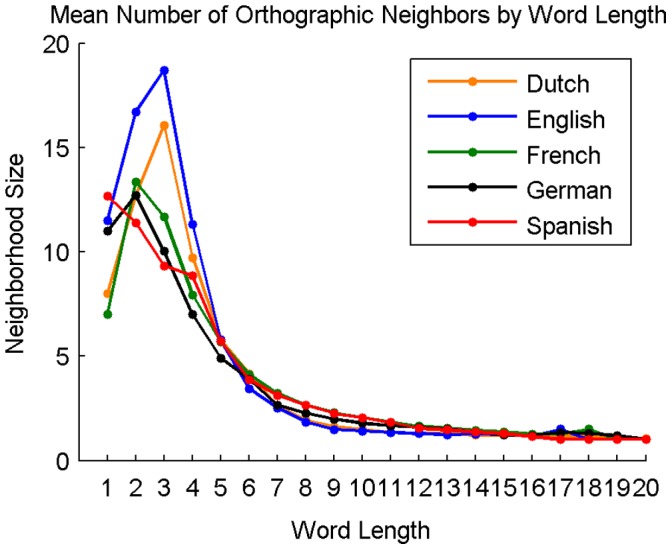
Average orthographic neighborhood size of words in Dutch, English, French, German, and Spanish at each word length.

### Calculating Neighborhoods

#### Orthographic neighborhoods

Orthographic neighbors consisted of words that differed only by the addition, deletion, or substitution of a single grapheme, as this method of calculating neighbors (including addition, deletion, and subtraction neighbors) provides a stronger metric of the lexical-level influence of neighborhood density than typical measures of substitution neighbors alone [3]. For example, the word *plant* has neighbors like *planet* (addition), *plan* (deletion), and *plank* (substitution). Likewise, the English word *chief* and the French word *chien* (meaning dog) are cross-linguistic orthographic neighbors because they differ only in the substitution of a single grapheme, “n” for “f.” Accented vowels and the Spanish “ñ” were treated as separate graphemes; therefore, words such as the French *ou* (English: ‘or’) and *où* (English: ‘where’) were considered to be orthographic neighbors.

**Figure 6:**
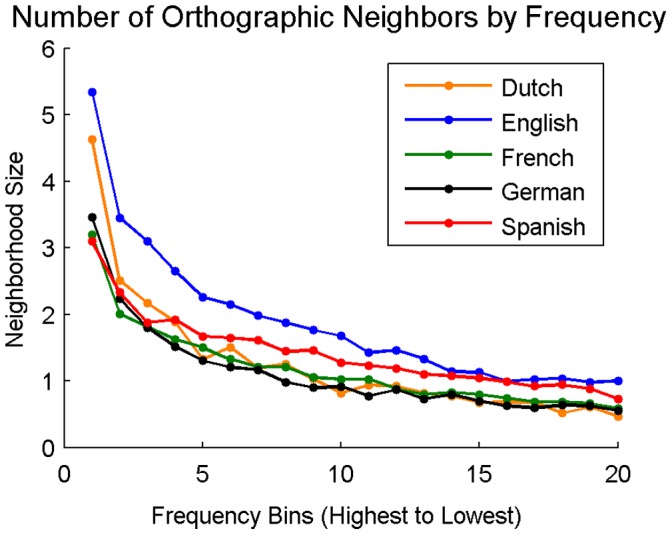
Average orthographic neighborhood size as a function of word frequency. Frequency bins are evenly spaced divisions of words in 5% increments. Bin one represents the average orthographic neighborhood size of the top 5% most frequent words in the language, bin twenty represents the average orthographic neighborhood size of the 5% least frequent words.

#### Phonological neighborhoods

Phonological transcriptions of each orthographic entry in the corpora were created using eSpeak (http://espeak.sourceforge.net/), an open-source text-to-speech software that provides IPA transcriptions for multiple languages. With this method, the phonological transcriptions of the corpora used machine-readable phonetic symbols based on the International Phonetic Alphabet so that language-to-language neighborhood comparisons were viable. To ensure the validity of eSpeak transcriptions, we selected a subset of words from each language that existed in both CLEARPOND and in a phonetic database for that language and calculated phonological neighborhoods (including substitution, addition, and deletion neighbors) for each word twice, once using the output provided by eSpeak and once using the output from the external database. The neighborhoods obtained by the two different metrics were very highly correlated: Dutch eSpeak comparison with the CELEX database [30]: *N* = 26,358, *R* = 0.94, *p*<0.001; English eSpeak comparison with the CMU database [31]: *N* = 26,474, *R* = 0.97, *p*<0.001; French eSpeak comparison with the Lexique database [26]: *N* = 27,751, *R* = 0.96, *p*<0.001; German eSpeak comparison with the CELEX database [30]: *N* = 21,609, *R* = 0.93, *p*<0.001; Spanish eSpeak comparison with the Busca Palabras database [8]: *N* = 10,978, *R* = 0.97, *p*<0.001. For examples of words in each language that correspond to each phoneme, see Table S1 and Table S2.

**Figure 7:**
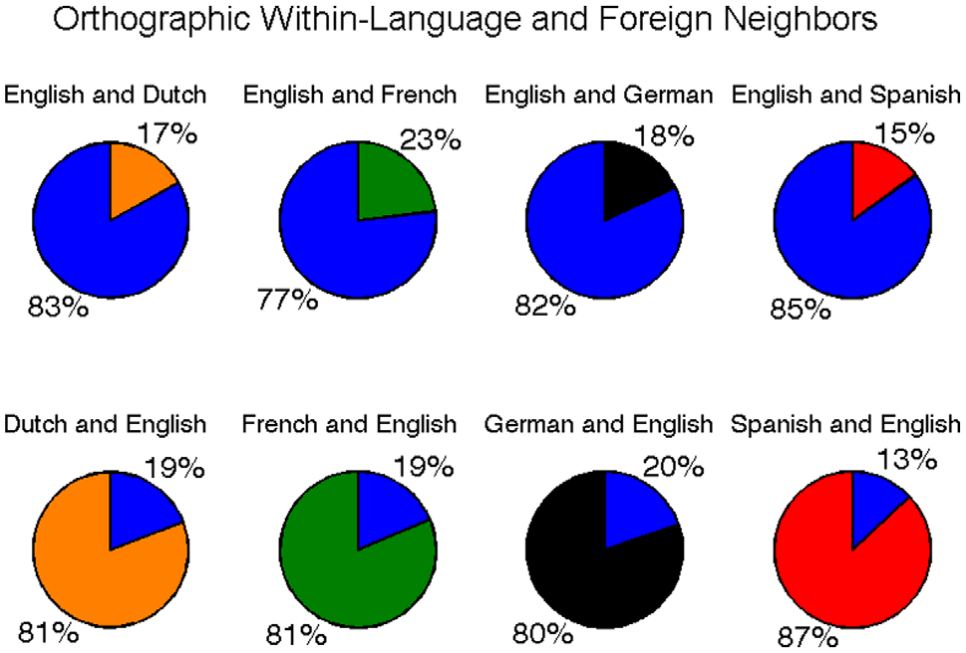
Ratio of within-language and foreign orthographic neighbors as part of total neighborhood size for each word with at least one neighbor. The top row compares the proportion of English within-language neighbors (blue) to foreign neighbors in each other language. The bottom row compares the proportion of within-language neighbors in each language to foreign (i.e., English) neighbors (blue).

**Table 1:** Mean orthographic within-language neighborhood size and foreign neighborhood size.

Language	Within-Language Neighborhood Size	Foreign Neighborhood Size				
		English	Dutch	French	German	Spanish
English	2.83 (0.03)	–	1.00 (0.02)	1.00 (0.01)	0.99 (0.01)	0.63 (0.01)
Dutch	2.00 (0.02)	1.00 (0.02)	–	–	–	–
French	2.35 (0.02)	1.00 (0.01)	–	–	–	–
German	1.97 (0.02)	0.99 (0.01)	–	–	–	–
Spanish	2.23 (0.02)	0.63 (0.01)	–	–	–	–

*Note*. Values represent means, those in parentheses represent standard error of the mean.

Phonological neighbors were composed of words that differed in the addition, deletion or substitution of a single phoneme [18], [32]. For instance, the English word *dough* (/doυ/) shares a neighborhood with words like *dome* (/doυm/; addition), *owe* (/oυ/; deletion), and *show* (/∫oυ/; substitution) in English. In addition, the English word *eel* (/il/) and the Spanish word *hilo* (/ilo/) are cross-linguistic neighbors by virtue of the deletion of the final phoneme/o/in the Spanish word.

Because the same subtitle corpora were used to calculate both orthographic and phonological neighborhoods, qualitative comparisons can be made across neighborhood types.

**Figure 8:**
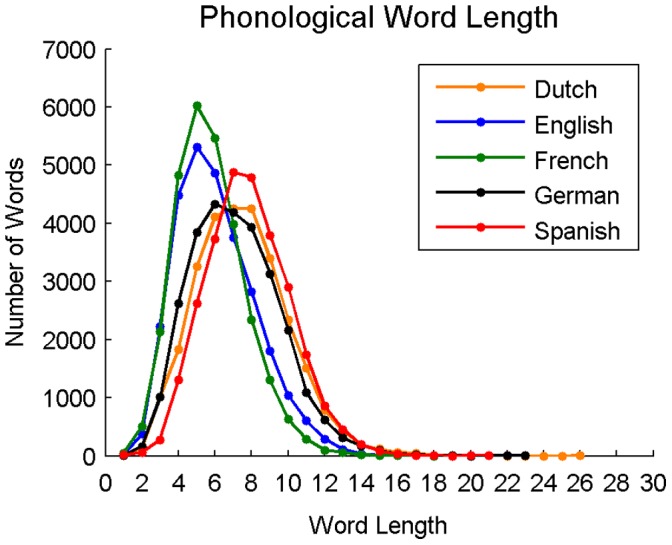
Distributions of phonological word lengths for Dutch, English, French, German, and Spanish.

#### Foreign neighborhoods

The same methods that were used to calculate orthographic and phonological neighborhoods within languages were used to calculate foreign neighbors. We calculated the Dutch, French, German, and Spanish neighbors of every English word, as well as the English neighbors of every Dutch, French, German, and Spanish word. For these analyses, the pool of candidate neighbors included all 27,751 words within the foreign language’s database. Because these foreign neighborhoods were constructed using the same databases used to calculate within-language neighborhoods, foreign and within-language neighborhoods of each language can be easily compared.

**Figure 9:**
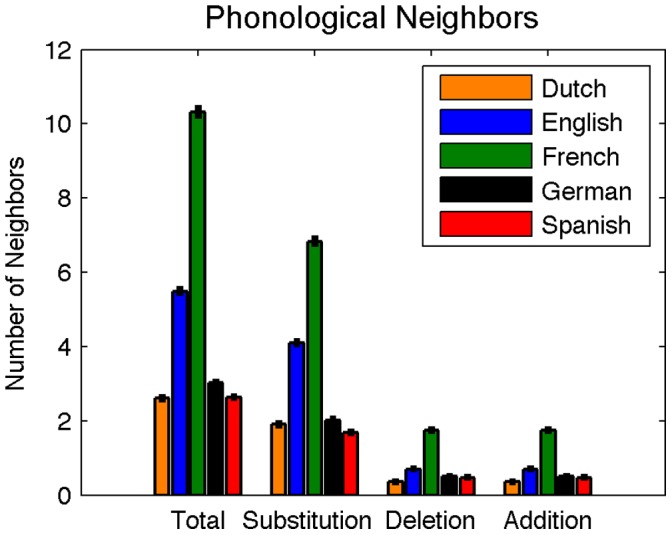
Mean phonological neighborhood sizes for words in Dutch, English, French, German, and Spanish. Total mean neighborhood size (left group) includes single-phoneme substitutions (e.g., ‘show’ for ‘dough’), deletions (e.g., ‘owe’ for ‘dough) and additions (e.g., ‘dome’ for ‘dough).

## Results

### Orthographic Neighborhoods

#### Orthographic word length

Average word length (in graphemes) was calculated for all 27,751 words in each language and was 8.41 (*SD* = 2.79) for Dutch, 7.26 (*SD* = 2.28) for English, 7.85 (*SD* = 2.26) for French, 8.25 (*SD* = 2.86) for German, and 7.94 (*SD* = 2.24) for Spanish; *F*(4,138750) = 879.66, *p*<0.001. Follow-up tests revealed that group differences were significant between all language pairs. The distribution of word lengths for each language is shown in Figure 2.

**Figure 10:**
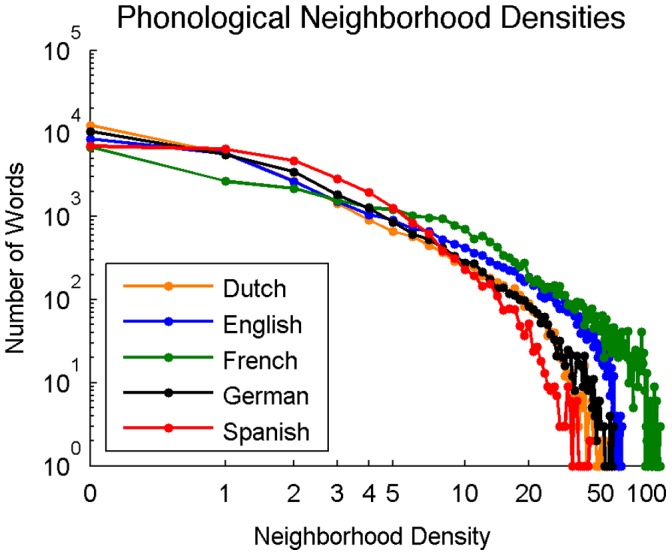
Distribution of phonological neighborhood densities across Dutch, English, French, German, and Spanish (log-log scale).

#### Orthographic neighborhood size

The number of within-language substitution, addition, and deletion neighbors was calculated for each word in each language. The mean neighborhood sizes are shown in Figure 3. For analysis purposes, the longest 5% of all words were collapsed into a single entry. An ANOVA with language and word length as factors revealed a significant effect of language on total orthographic neighborhood size, *F*(4,138690) = 12.69, *p*<0.0001, a significant effect of word length *F*(12,138690) = 9829.49, *p*<0.0001, and a significant language x word length interaction *F*(48,138690) = 222.25, *p*<0.0001. Post-hoc comparisons on the estimated marginal means for language revealed that English words contained significantly more neighbors than words in Dutch, French, German, or Spanish (all *p*’s <0.05).

While the effect of substitution neighbors on linguistic processing has long been studied, recent evidence suggests that addition and deletion neighbors affect word processing as well [3]. To best characterize the effect of orthographic neighbors on word processing, all further analyses will consider the sum total of substitution, deletion, and addition neighbors for each word.

**Figure 11:**
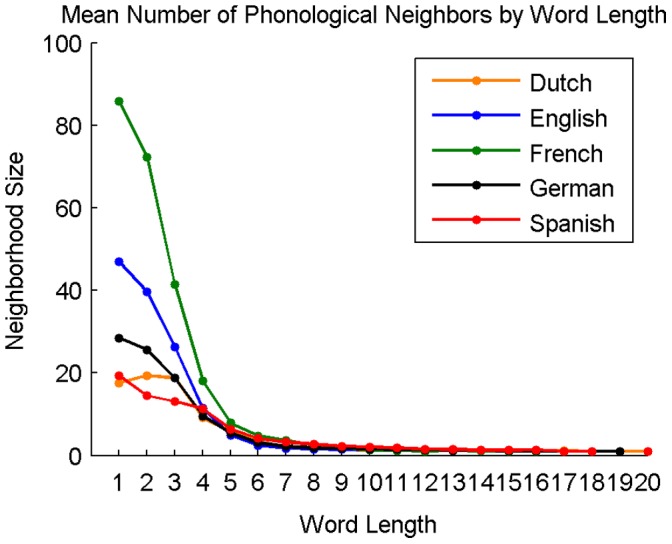
Average phonological neighborhood size of words in Dutch, English, French, German, and Spanish at each word length.

**Figure 12:**
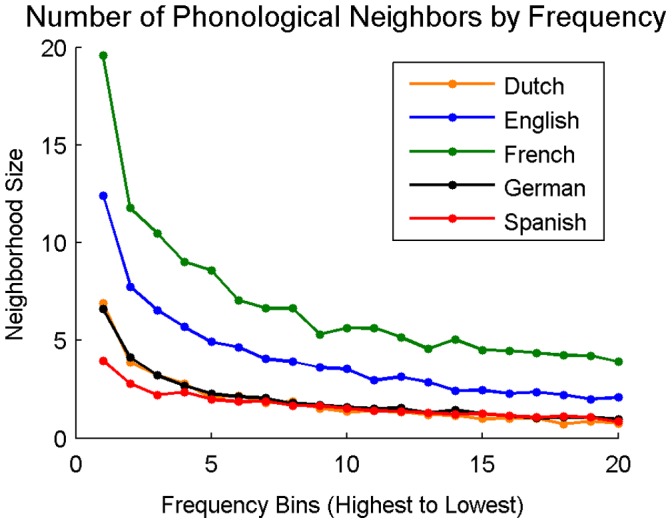
Average phonological neighborhood size as a function of word frequency. Frequency bins are evenly spaced divisions of words in 5% increments. Bin one represents the average phonological neighborhood size of the top 5% most frequent words in the language, bin twenty represents the average phonological neighborhood size of the 5% least frequent words.

**Table 2:** Mean phonological within-language and foreign neighborhood size.

Language	Within-Language Neighborhood Size	Foreign Neighborhood Size				
		English	Dutch	French	German	Spanish
English	5.49 (0.06)	–	0.89 (0.02)	1.23 (0.04)	0.89 (0.02)	0.15 (0.01)
Dutch	3.05 (0.04)	0.89 (0.02)	–	–	–	–
French	10.32 (0.10)	1.23 (0.04)	–	–	–	–
German	3.02 (0.03)	0.89 (0.02)	–	–	–	–
Spanish	2.63 (0.02)	0.15 (0.01)	–	–	–	–

*Note*. Values represent means, those in parentheses represent standard error of the mean.

**Figure 13:**
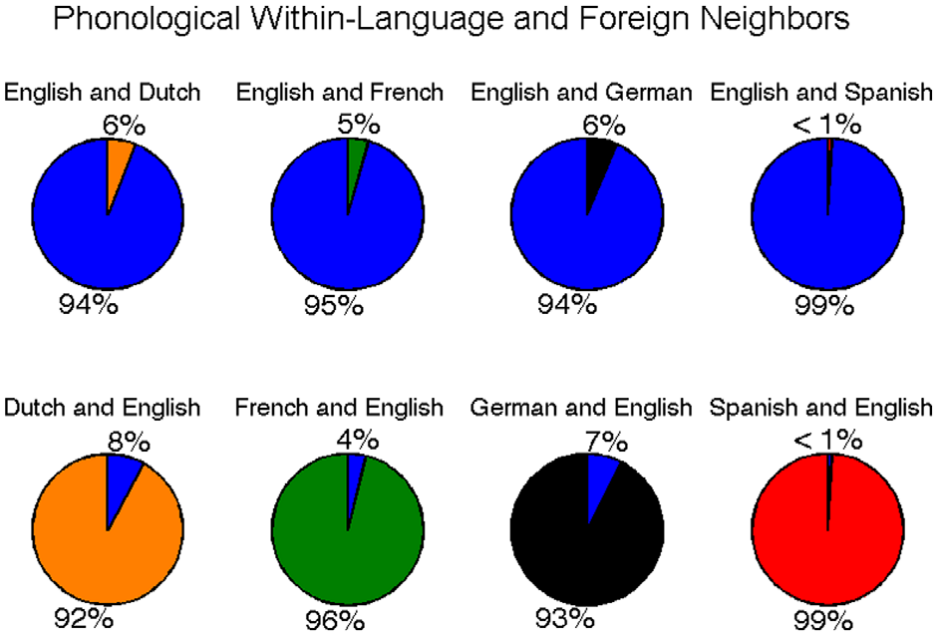
Ratio of within-language and foreign phonological neighbors as part of total neighborhood size for each word. The top row compares the proportion of English within-language neighbors (blue) to foreign neighbors in each other language. The bottom row compares the proportion of within-language neighbors in each language to foreign (i.e., English) neighbors (blue).

**Figure 14:**
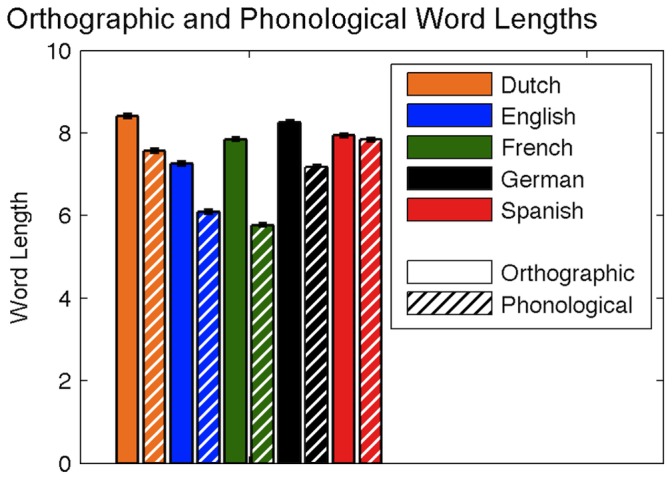
Comparisons of orthographic and phonological word lengths for Dutch, English, French, German, and Spanish.

#### Distribution of orthographic neighborhood densities

Figure 4 shows the distribution of neighborhood densities across languages. The percentage of words in each language with at least one orthographic neighbor was 55.3% for Dutch, 64.1% for English, 77.2% for French, 61.0% for German, and 74.7% for Spanish.

#### Orthographic neighborhood size by word length

Figure 5 shows the average neighborhood size of words in each language for each word length.

#### Orthographic neighborhood size by word frequency

In each language, all 27,751 words were divided into twenty equally spaced frequency bins, with each bin representing a 5% increment. For example, bin one represented the average orthographic neighborhood size of the top 5% most frequent words in the language while bin 20 represented the average orthographic neighborhood size of the least frequent 5% of words. The average orthographic neighborhood size for words in each of these frequency bins is provided in Figure 6.

#### Foreign orthographic neighbors

Foreign orthographic neighborhoods were calculated for each English word in Dutch, French, German, and Spanish, and for each Dutch, French, German, and Spanish word in English. Results revealed that 21.2% of English words had at least one Dutch neighbor, 31.7% had at least one French neighbor, 23.6% had at least one German neighbor, and 21.7% had at least one Spanish neighbor. In addition, 28.0% of Dutch words, 33.9% of French words, 30.0% of German words, and 22.8% of Spanish words had at least one English neighbor. The effect of foreign neighbors on orthographic neighborhood size is provided in Table 1. For each word with at least one within-language or foreign neighbor, the relative proportion of neighbors to all of a word’s neighbors was calculated. Mean proportions are provided in Figure 7.

**Figure 15:**
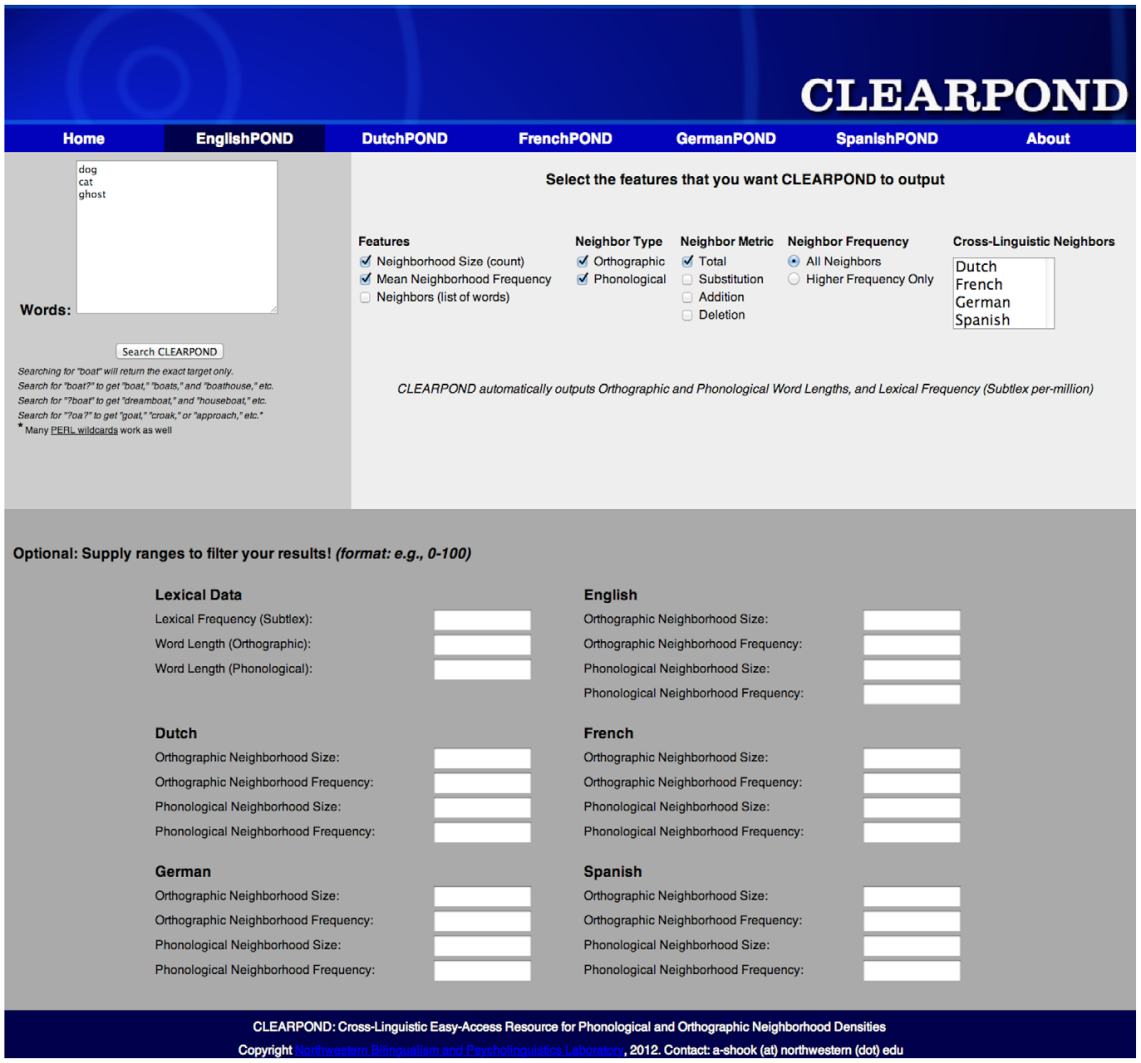
Screen-shot of the EnglishPOND portion of the CLEARPOND website, accessible at http://clearpond.northwestern.edu **. CLEARPOND provides a user-friendly, web-based interface for obtaining Dutch, English, French, German, and Spanish phonological and orthographic neighborhood densities (or, PONDs).** The search function allows users to search for POND information in any of the five languages using single word queries or by providing full lists of words. CLEARPOND provides a number of important psycholinguistic measures, such as neighborhood density and neighborhood frequency, both for within-language neighbors and foreign-language neighbors. With user-controlled output selection, researchers can choose the output parameters that are most relevant. In addition to allowing users to acquire data for specific words, CLEARPOND can also search by features so that researchers can generate new lists of words that meet precise criteria, such as a specific range of neighborhood sizes or lexical frequency (as provided by the Subtlex databases). Furthermore, multiple filters can be applied simultaneously, providing greater control for stimuli creation. Users also have the option of exporting their results directly to a text file, making it easy to create downloadable documents containing pertinent psycholinguistic measures for all of their stimuli. In addition to the web-based interface, more comprehensive lists containing all of the information provided by the database are available for download, so that the entire CLEARPOND database can be accessed offline.

### Phonological Neighborhoods

#### Phonological word length

Average word length (in phonemes) was calculated for all 27,751 words in each language and was 7.48 (*SD* = 2.51) for Dutch, 6.09 (*SD* = 2.18) for English, 5.77 (*SD* = 1.93) for French, 7.14 (*SD* = 2.45) for German, and 7.84 (*SD* = 2.28) for Spanish; *F*(4,138750) = 4284.86, *p*<0.001. Follow-up tests revealed that group differences were significant between all language pairs. The distribution of word lengths for each language is shown in Figure 8.

#### Phonological neighborhood size

The number of within-language substitution, addition, and deletion neighbors was calculated for each word in each language. The mean neighborhood sizes are shown in Figure 9. For analysis purposes, the longest 5% of all words were collapsed into a single entry. An ANOVA with language and word length as factors revealed a significant effect of language on total phonological neighborhood size, *F*(4,138695) = 2730.64, *p*<0.0001, a significant effect of word length *F*(11,138695) = 10204.84, *p*<0.0001, and a significant language x word length interaction *F*(44,138695) = 913.84, *p*<0.0001. Post-hoc comparisons on the estimated marginal means for language revealed that all languages differed on phonological neighborhood size (all *p*’s<0.05). As in the orthographic neighborhood analyses, all further phonological neighborhood analyses consider the total number of substitution, addition, and deletion neighbors for each word.

#### Distribution of phonological neighborhood densities

Figure 10 shows the distribution of phonological neighborhood densities across languages. The percentage of words in each language with at least one neighbor was 55.2% for Dutch, 69.1% for English, 75.5% for French, 61.9% for German, and 74.6% for Spanish.

#### Phonological neighborhood size by word length

Figure 11 shows the average neighborhood size in each language for each word length.

#### Phonological neighborhood size by word frequency

In each language, all 27,751 words were divided into twenty equally spaced frequency bins (as was done with orthographic neighborhoods). The average phonological neighborhood size for words in each frequency bin is provided in Figure 12.

#### Foreign phonological neighbors

Foreign phonological neighborhoods were calculated for each English word in Dutch, French, German, and Spanish, and for each Dutch, French, German, and Spanish word in English. Results revealed that 15.9% of English words had at least one Dutch neighbor, 10.6% had at least one French neighbor, 15.8% had at least one German neighbor, and 4.8% had at least one Spanish neighbor. In addition, 10.8% of Dutch words, 12.0% of French words, 12.4% of German words, and 1.6% of Spanish words had at least one English neighbor. The effect of foreign neighbors on phonological neighborhood size is provided in Table 2. For each word with at least one within-language or foreign neighbor, the relative proportion of within-language or foreign neighbors to all of a word’s neighbors was calculated. Mean proportions are provided in Figure 13.

## Discussion

The results of our analyses show consistent patterns across languages in the effects of word length and lexical frequency on neighborhood size. Differences across languages are also present – for example, while French has the most phonological neighbors, English contains more orthographic neighbors than the other four languages examined. The degree of similarity between phonological and orthographic neighbors also varies across languages (e.g., in Spanish, phonological and orthographic neighborhoods are more alike than in any other language). Within languages, differences emerge dependent on neighborhood origin; foreign neighbors are relatively infrequent compared to within-language neighbors.

### Comparing Orthographic and Phonological Neighborhoods

Because the present analysis derived orthographic and phonological neighborhoods from the same subtitle corpora, we were able to make direct comparisons between the two neighborhood types. The differences that emerge in the relationships between these neighborhoods across languages can potentially be used to illuminate differences in language transparency. Transparency, or orthographic depth, is a measure of how closely a language maintains a one-to-one grapheme-phoneme correspondence; the more transparent a language, the more the graphemes and phonemes are tightly matched. For example, in the most transparent of languages, each phoneme would map to only one grapheme and vice versa (e.g., the Spanish phoneme/m/is always represented by the grapheme *m*, and the *m* grapheme always corresponds to the phoneme/m/). Conversely, opaque languages are those in which grapheme-phoneme mappings are less consistent; multiple graphemes can represent the same phoneme (e.g., English *k* and *c* can both represent the phoneme/k/), and more than one phoneme may be represented by a single grapheme (e.g., English *g* can represent the phonemes/g/and/ 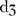/). Because the grapheme-phoneme mappings of transparent languages are consistent, in these languages, many orthographic neighbors are also phonological neighbors. When phonemes and graphemes are consistently matched, the phonetic transcriptions of words mirror the orthographic structure. Therefore, when a single *grapheme* substitution (or addition or deletion) results in the creation of a new word, it is likely that the new word similarly differs from the original in only one *phoneme*.

Our analyses suggest that, in addition to indexing language transparency as a strict match between grapheme-phoneme correspondences, there may be a relationship between a language’s transparency and the degree of similarity between the language’s orthographic and phonological neighborhoods. For example, Spanish and German (both considered to be transparent languages [33]), demonstrate a high degree of similarity in the distributions of their orthographic and phonological neighborhoods. However, the similarity between orthographic and phonological neighborhoods is not quite as tightly coupled in German as it is in Spanish, likely because, German contains specific consonant clusters (e.g., *sch*) that correspond to single phonemes (e.g.,/∫/). Accordingly, there is higher similarity between graphemic and phonemic word lengths in Spanish than in German, Dutch, or English (Figure 14). French, a language with a high number of silent letters and digraphs, has the largest difference between graphemic and phonemic word length.

### Comparing Types of Neighbors

In addition to revealing differences between phonological and orthographic neighborhoods, our data illustrate differences in how substitution, addition, and deletion neighbors are used across languages.

#### Orthographic neighborhoods

Relative to the other four languages, English contains a large number of orthographic substitution neighbors. This suggests that English makes use of more available letter sequences at every word length, and efficiently uses its graphemic space. In contrast, French derives a greater percentage of its neighbors from addition and deletion relative to the other languages. Although French has relatively few substitution neighbors, it nevertheless has the second largest total number of neighbors; this is driven by French’s increased use of addition and deletion neighbors.

#### Phonological neighborhoods

A notable trend that emerged in the comparison of phonological neighborhood sizes across languages is the much higher occurrence of phonological neighbors of all types (substitution, deletion, and addition) in French when compared to all other languages. One potential explanation for the observed trend is the large number of homophones in the French language.

Homophones increase the phonological neighborhood density of a language because there are multiple lexical entries with the same phonological make-up. Therefore, if a word has a phonological neighbor that is one meaning of a homophonic word set, it also automatically has a phonological neighbor comprised of all other homophones. In languages such as French, where homophonic word sets are numerous, the phonetic diversity of all tokens is decreased, and the pool of potential phonological neighbors is increased. For example, the French word *mer* (sea) is a substitution neighbor of *ver* (earthworm), *vers* (towards), *vert* (green), and *verre* (drinking glass), which are all pronounced/vε<$>\raster(80%)="rg3"<$>/; only *ver* would be an orthographic neighbor. The homophone account of French’s increased phonological neighborhood density is consistent with an analysis of phonetic diversity across languages: French only contained 17,303 unique phonetic words (out of 27,751; 62.4%), compared to 27,258 in Dutch (98.0%), 27,007 in English (97.3%), 27,284 in German (98.3%), and 27,101 in Spanish (97.7%).

### Foreign Neighborhoods

In our analysis of foreign neighbors, we restricted comparisons to English and each other language (Dutch, French, German, and Spanish) to facilitate ease of comparisons, and because English is one of the most commonly learned second languages [34]. Foreign orthographic neighbors were found to make relatively substantial contributions to overall neighborhood size, constituting between 13–20% of a word’s total neighbors on average. Within-language neighbors still dominated overall neighborhood size, likely because languages have different orthotactic rules and requirements for the formulation of valid words. The result is that words in each of the languages we examined were more similar in orthographic form to other words within the same language than they were to foreign words.

Compared to foreign orthographic neighbors, foreign phonological neighbors were very rare. The effect of foreign phonological neighbors on overall neighborhood size was quite low, and the percentage of a word’s neighbors that derived from a foreign language was even lower, between 1–8%. These results are consistent with those of Vitevitch [32], who conducted an analysis of foreign phonological neighbors across Spanish and English and found that the two languages share relatively few neighbors.

One potential reason for the small number of foreign neighbors is that though the five languages we investigated share an alphabetic system (aside from accented letters), they contain phonological systems that are much more distinct. Because the orthographic structure of a language is anchored by that language’s writing system, orthography does not vary much over time. Conversely, a language’s phonetic structure has much more freedom to vary over time and across geographical space; the accumulation of these phonological changes likely contributes to the languages’ phonological distinctiveness, thereby reducing the number of foreign phonological neighbors.

While comparisons of foreign neighbors can be used for purposes of stimuli construction and to validate cross-linguistic comparisons, it is important to note that our data should not be interpreted as a measure of the bilingual mental lexicon. In order to make true claims about the nature of bilingual lexical representations based on corpus analyses, it would first be necessary to procure a bilingual corpus in which frequency values are representative of usage when a single individual speaks two languages. To our knowledge, such a corpus does not exist. If bilingual corpora can be obtained, it would be worthwhile to conduct neighborhood analyses using those lexical entries.

### Conclusions and Future Directions

The corpus analysis presented in the current study provides a novel tool for researchers who study language processing. It enables comparisons between orthographic and phonological neighbors and within and across five languages.

While neighborhood information for some languages has been made available in the past [13], [15], [20], [21], the database that we present here provides comparable corpora and analyses across languages. We also expand upon the past examinations of foreign neighbors in Spanish and English [32] by supplying foreign neighborhood data for four language pairs – English-Dutch, English-French, English-German, English-Spanish – and by including both orthographic and phonological neighbors. Our future efforts will focus on developing a comparable corpus derived from written word data using written-word databases, such as Google Ngram (http://books.google.com/ngram) to complement our present work on spoken language.

In sum, the current paper presents a unified database for indexing neighborhood information derived from spoken corpora. These data provide cross-linguistic metrics that are crucial for designing experiments of spoken and written language processing. We have made our database available in searchable form (see Figure 15 for a screenshot of the web interface) at http://clearpond.northwestern.edu; it is also freely available for download.

## Supporting Information

Table S1 **IPA consonants and example words in each language.**
(DOCX)

Table S2 **IPA vowels and example words in each language.**
(DOCX)
