# Supplementary material for: CLEARPOND: Cross-Linguistic Easy-Access Resource for Phonological and Orthographic Neighborhood Densities
Source: PLoS One. 2012 Aug 20;7(8):e43230. doi: 10.1371/journal.pone.0043230 (PMC3423352; doi:10.1371/journal.pone.0043230)
Supplement: Table S1 — IPA consonants and example words in each language. (DOCX) [file pone.0043230.s001.docx]

*Table S1.* IPA consonants and example words in each language

| **IPA Symbol** | **Dutch** | **English** | **French** | **German** | **Spanish** |
| --- | --- | --- | --- | --- | --- |
| b | he**bb**en | **b**ut | **b**ien | ha**b**en | **b**año |
| β | - | - | - | - | fa**v**or |
| ç | - | - | - | i**ch** | - |
| d | **d**at | o**dd** | **d**eux | **d**as | **d**el |
| dʒ | bu**dg**et | **j**ob | léthar**g**ie | mana**g**er | - |
| f | hee**f**t | **f**or | **f**aire | **v**on | **f**avor |
| g | zo**g**enaamde | **g**et | **g**rand | sa**g**en | **g**racias |
| ɣ | **g**een | - | - | - | se**g**uir |
| h | **h**ij | **h**at | - | **h**ast | - |
| j | **j**aar | **y**ou | fi**ll**e | **j**a | d**io**s |
| k | i**k** | **c**an | **qu**i | **k**ann | **qu**e |
| l | we**l** | **l**ike | **l**oup | **l**eute | fe**l**iz |
| ʎ | - | - | - | - | si**ll**a |
| m | **m**aar | **m**e | **m**ais | **m**it | **m**ucho |
| n | **n**ek | **n**eed | **n**ous | **n**icht | **n**ada |
| ŋ | jo**ng** | goi**ng** | parki**ng** | la**ng** | ci**n**co |
| ɲ | ora**ng**je | - | si**gn**e | - | se**ñ**or |
| p | **p**raat | **p**ut | **p**our | **p**roblem | **p**ara |
| pf | - | - | - | **pf**erd | - |
| r | p**r**aat | - | - | - | gue**rr**a |
| ɾ | - | - | - | - | p**r**oblema |
| ʁ | - | - | t**r**ès | f**r**au | - |
| ɹ | - | **r**ight | - | - | - |
| s | men**s**en | **s**ome | **s**uis | i**s**t | lo**s** |
| ∫ | so**ci**aal | **sh**e | **ch**ez | **sch**ön | - |
| t | he**t** | **t**ime | **t**out | mi**t** | **t**odos |
| ts | - | - | - | **z**u | - |
| t∫ | **ch**ecken | whi**ch** | mat**ch** | deu**tsch**e | mu**ch**o |
| θ | - | **th**ink | - | - | ha**c**er |
| ð | - | **th**at | - | - | na**d**a |
| v | **v**oor | **v**ery | a**v**ec | **w**as | - |
| ʋ | **w**aarom | - | - | - | - |
| w | be**w**aren | **wh**at | **ou**i | - | b**u**eno |
| x | to**ch** | lo**ch** | x | au**ch** | di**j**o |
| z | **z**ijn | ha**s** | be**s**oin | **s**ehr | - |
| ʒ | vi**s**ioen | plea**s**ure | **j**e | passa**g**iere | - |
