# Supplementary material for: CLEARPOND: Cross-Linguistic Easy-Access Resource for Phonological and Orthographic Neighborhood Densities
Source: PLoS One. 2012 Aug 20;7(8):e43230. doi: 10.1371/journal.pone.0043230 (PMC3423352; doi:10.1371/journal.pone.0043230)
Supplement: Table S2 — IPA vowels and example words in each language. (DOCX) [file pone.0043230.s002.docx]

*Table S2.* IPA vowels and example words in each language

| **IPA Symbol** | | **Dutch** | **English** | **French** | **German** | **Spanish** |
| --- | --- | --- | --- | --- | --- | --- |
| **Vowels** | | | | | | |
| æ | | - | c**a**t | - | - | - |
| a | | d**a**t | - | p**a**s | w**a**s | m**á**s |
| aː | | n**aa**r | - | - | - | - |
| ɑ | | - | f**a**ther | - | - | - |
| e | | - | - | all**er** | - | hombr**e** |
| ɛ | | g**e**ld | g**e**t | c**e**tte | d**e**nn | p**e**ro |
| ɛː | | - | - | - | sp**ä**ter | - |
| ə | | onz**e** | comm**a** | p**e**tit | bitt**e** | - |
| i | | n**i**et | n**ee**d | qu**i** | l**ie**be | s**í** |
| ɪ | | d**i**t | w**i**th | - | m**i**t | - |
| Ï | | - | an**y**thing | - | - | - |
| œ | | l**eu**k | - | p**eu**r | k**ö**nnen | - |
| ø | | - | - | v**eu**x | sch**ö**n | - |
| o | | - | - | v**o**tre | **o**der | h**o**la |
| ɔ | | t**o**ch | **o**ff | - | n**o**ch | - |
| ɔː | | s**oo**rt | - | - | - | - |
| u | | g**oe**d | y**ou** | v**ou**s | g**u**te | l**u**gar |
| ʊ | | - | p**u**t | - | **u**nd | - |
| ʌ | | - | b**u**t | - | - | - |
| y | | b**uu**rt | - | t**u** | **ü**ber | - |
| yː | | d**uw** | - | - | f**ü**r | - |
| ʏ | | h**u**t | - | - | - | - |
| **Dipthongs** | | | | | | |
| aɪ | | - | m**y** | - | **ei**n | b**ai**le |
| aʊ | | - | n**ow** | - | fr**au** | p**au**sa |
| eɪ | | - | th**ey** | - | - | s**ei**s |
| ɛi | | h**ij** | - | - | - | - |
| œy | | t**ui**n | - | - | - | - |
| oʊ | | b**oo**t | kn**ow** | - | - | - |
| ɔɪ | | - | b**oy** | - | - | est**oy** |
| ɔʏ | | - | - | - | l**eu**te | - |
| ʌu | | j**ou** | - | - | - | - |
| **Nasal Vowels** | | | | | | |
| ɑ̃ | | - | - | d**an**s | - | - |
| ɛ̃ | | - | - | bi**en** | - | - |
| œ̃ | | - | - | auc**un** | - | - |
| ɔ̃ | | - | - | n**on** | - | - |
| **Rhotic Vowels** | | | | | | |
| ɝ | - | | h**er** | - | - | - |
| ɚ | - | | nev**er** | - | ab**er** | - |
| ɑɹ | - | | c**ar** | - | - | - |
| ɛɹ | - | | th**er**e | - | - | - |
| iəɹ | - | | n**ear** | - | - | - |
| oɹ | - | | f**or** | - | - | - |
| ʊɹ | - | | t**our** | - | - | - |
